# Predicting missing links in complex networks based on common neighbors and distance

**DOI:** 10.1038/srep38208

**Published:** 2016-12-01

**Authors:** Jinxuan Yang, Xiao-Dong Zhang

**Affiliations:** 1 School of Mathematical Science, MOE-LSC, SHL-MAC, Shanghai Jiao Tong University, 800 Dongchuan Road, Shanghai, 200240, P.R. China

## Abstract

The algorithms based on common neighbors metric to predict missing links in complex networks are very popular, but most of these algorithms do not account for missing links between nodes with no common neighbors. It is not accurate enough to reconstruct networks by using these methods in some cases especially when between nodes have less common neighbors. We proposed in this paper a new algorithm based on common neighbors and distance to improve accuracy of link prediction. Our proposed algorithm makes remarkable effect in predicting the missing links between nodes with no common neighbors and performs better than most existing currently used methods for a variety of real-world networks without increasing complexity.

As an important branch of network data analysis, predicting missing links in complex network has attracted many researchers’ widespread attentions not only because data collected from network platforms is incomplete^1^,^2^, but also it is helpful to understand evolution of networks^3^,^4^,^5^, and predict future conflict and individual preferences^6^,^7^. In principle many evolution models correspond to a link prediction approach. Thus, link prediction can be used in revealing hidden information and evaluating the performance of distinct models. Moreover, link prediction has been applied to health care and communication field to identify abnormal cases^8^,^9^.

The main work in link prediction is to estimate the missing link between two nodes based on current links and interactions in networks^10^. Link prediction discusses missing links and spurious links. In this paper, we focus on predicting missing links. Generally, between nodes with very high likelihood scores are considered to be highly likely to have missing links. In the past few years, many prediction methods based on topological structure of networks have been proposed related to local paths, common neighbors and random walk^10^,^11^,^12^,^13^. In social networks two individuals who have more common friends are very likely to be friends in future. Furthermore, community methods, hierarchical models and probabilistic methods are also used for link prediction^14^,^15^,^16^,^17^,^18^,^19^. Recently, information theory and spectral method of adjacency matrix have been adopted to find missing links^20^,^21^,^22^.

The prediction methods based on common neighbors metric^10^ are very popular due to its simplicity. But with single common neighbors metric is not accurate enough to reveal the similarities between nodes and reconstruct properly networks, especially there are less common neighbors between nodes in sparse networks. A part of missing links could not be predicted because there are no common neighbors between them, but they often play a key role to connect different communities, and affect network properties, such as betweenness centrality, average distance, congestion and spreading ability. Therefore, it is important to propose an algorithm to predict missing links between nodes with no common neighbors.

A lot of real-world networks indicate high clustering properties. There are a large number of short loops. It is a good idea to exploit this properties to improve accuracy of link prediction. In this paper, we separate link prediction into two parts: predicted links that generate loops of length 3 and predicted links that generate short loops of length more than 3. So common neighbors and distance metric are adopted to predict these loops. The key question is to estimate the proportion of two parts. A new method is proposed to achieve it. By estimating the proportion of missing links between nodes with no common neighbors in total missing links, our algorithm makes remarkable effect to predict the missing links between nodes with no common neighbors, and improve the accuracy of link prediction. The experimental results show that it can obtain significantly better prediction accuracy for a variety of real-world networks than other methods.

## Results

### Data sets Description

The test data sets of real-world networks in this paper are:

- Karate: The test data of Karate club network was collected by Zachary, which indicates the interactions of 34 members of a university Karate club^23^.
- Dolphins: It is an animal relationship network studied by Lusseau *et al*. with 62 bottlenose dolphins living in Doubtful Sound of New Zealand^24^.
- Polbook: This is a network of books about US politics published around the time of the 2004 presidential election and sold by the online bookseller Amazon.com. The network was compiled by Krebs and is unpublished, but can be found on Krebs’ website (see http://www.orgnet.com).
- Word: The data is a network of common adjective and noun adjacencies for the novel “David Copperfield” by Charles Dickens, as described by Newman^25^.
- Neural: This data represents the neural network of C. Elegans. The nodes in the original data are not consecutively numbered, so they have been renumbered to be consecutive^26^.
- Circuit: Electronic circuits can be viewed as networks in which nodes are electronic components (like capacitors, diodes, etc.) and connections are wires. Our network maps one of the benchmark circuits of the so-called ISCAS'89 set (see data set from http://www.weizmann.ac.il/mcb/UriAlon/)^27^.
- Email: This is a network of e-mail interchanges between members of the Universitat Rovira i Virgili (Tarragona) (see data set from http://deim.urv.cat/~alexandre.arenas/data/welcome.htm)^28^.
- Power: This is an undirected, unweighted network representing the topology of the Western States Power Grid of the United States^26^.

The data sets (1–5) and (8) can be downloaded from Mark Newman’s network data sets: http://www-personal.umich.edu/~mejn/netdata/. The parameters of networks about the number of nodes *N*, the number of links *m*, average degree 〈*k*〉, average distance 〈*d*〉, assortativity coefficient *r* and degree heterogeneity *H* are listed in Table 1.

### Link prediction method

Two metrics are used to evaluate the accuracy of link prediction methods: *AUC* (areas under the receiver operating characteristic curve)^29^ and *Precision*^30^. Given an unweighted and undirected network *G* = (*V*, *E*) with vertex set *V* = {*v*_1_, *v*_2_, …, *v*_*N*_} and the observed link set *E*, where the size of *E* is *m*. The self-loops and multiple links are not allowed. *E* are randomly divided into two disjoint subsets: the probe set *E*^*P*^ and the training set *E*^*T*^. *E*^*P*^ is used for testing and is viewed as unknown information. *E*^*T*^ is viewed as known information. A good prediction method should have high *AUC* value according to the definition of *AUC*, i.e. the links in probe set have higher scores than non-existing links. *Precision* is computed as the fraction of correct predicted links in the top-*L* ranking lists, where *L* is the total number of missing links (*L* = |*E*^*P*^|) (see Methods section).

Now *G*′ = (*V*, *E*^*T*^) is known, so basic idea in reconstructing network is to add top-*L* predicted links to *G*′ to obtain G^*^ = (*V*, E^*^) so that G^*^ is as close as possible to *G*. Therefore, a good predicted method can provide trusted prediction in the evolution of networks.

In this paper we separate the probe set *E*^*P*^ into two subsets: 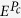 and 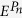, which denote link set between nodes with common neighbors and link set between nodes with no common neighbors in *G*′, respectively, that is,


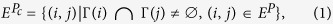



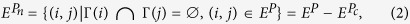


where Γ(*i*) denotes the set of neighbors of node *i*. Let 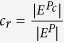 is the proportion of 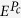 in *E*^*P*^. The test will be performed with *E*^*P*^ accounting for 10% of the observed link set *E*, and randomly selects *E*^*P*^ to remove every time. The results of *c*_*r*_ are the average of 20 realizations for each network (see Table 1).

But for a practical observed network *G*, link prediction methods are used to predict the possible links in the future (network evolution), we only knew roughly the total number of missing links *L* = |*E*^*P*^|, which is consistent with other methods in literatures. Therefore, it is key to estimate the proportion of 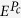 in *E*^*P*^ in order to improve prediction accuracy under the *Precision* metric. We define 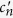 to evaluate strength of links between nodes with common neighbors in *G*′ = (*V*, *E*^*T*^) (The corresponding definition is *c*_*n*_ for *G*, and *c*_*n*_ is labeled as “CN coefficient” in this paper), which is defined as the fraction of links between nodes with common neighbors in *E*^*T*^, that is,


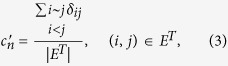



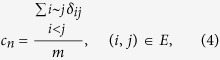


where *δ*_*ij*_ = 1, if Γ(*i*) ∩ Γ(*j*) ≠ ∅, 0 otherwise, and *i* ~ *j* denotes node *i* and *j* to be adjacent. The results of 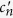 and *c*_*n*_ are listed in Table 1. Between 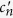 and *c*_*r*_ has low RMSE (root-mean-square error) and high positive correlation measured by Pearson correlation coefficient (*CC*). Furthermore, between *c*_*n*_, *c*_*r*_ and clustering coefficient *c* (defined in Methods section) also indicate high positive correlation (see Table 2). Therefore, it is feasible to use 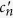 instead of *c*_*r*_ to estimate the proportion of 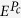 in *E*^*P*^. Thus, 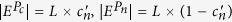 in *G*′.

**Figure 1:**
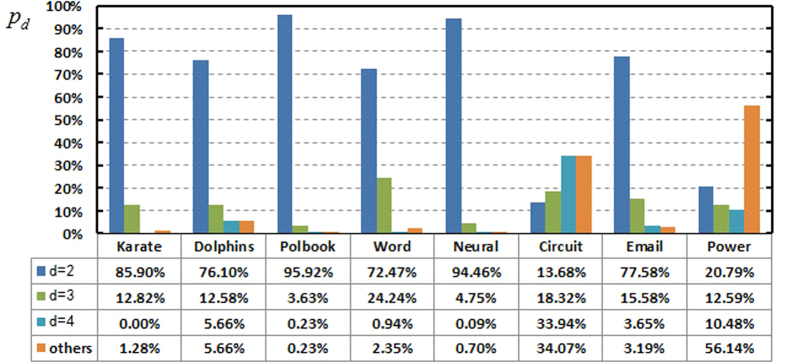
The distributions *p*_*d*_ for d = 2, 3, 4 and the rest in 8 real-world networks.

There are large number of short loops in real-world networks. In order to illustrate the fact, the distribution of “pseudo-distance” will be given. Generally, the distance *d*_*ij*_ of two nodes *i*, *j* is defined as the length of the shortest paths between them. *d*_*ij*_ is infinite if no such path exist. “pseudo-distance” 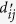 is defined to be the length of the shortest paths between *i* and *j* in network *G* − *e* for a link *e* = (*i*, *j*), that is, 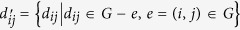. Let


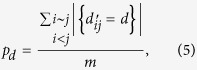


which denotes the fraction of links where pseudo-distance is *d* in total links. In a way it can reveal distance distribution of missing links in *G*′. Figure 1 describes the distribution *p*_*d*_ for d = 2, 3, 4 and the rest in 8 real-world networks, where the distribution of most networks are concentrated in *d* = 2, 3, 4. It is obvious that *p*_2_ = *c*_*n*_.

On the other hand, these missing links for *d* ≠ 2 (links between nodes with no common neighbors) play a pivotal role in determining the structure properties of networks. But they are neglected by mostly current existing link prediction methods. A single method based on common neighbors could not predict these important missing links. So we propose in this paper a new prediction method based on common neighbors and distance. The score of likelihood is defined as follows:


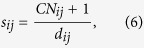


where *CN*_*ij*_ = |Γ(*i*) ∩ Γ(*j*)| represents the number of common neighbors for node *i* and *j*. The above equation equals to:


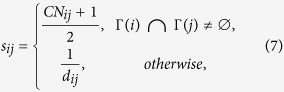


Since between two nodes is more likely to possess a link if they have more common neighbors, it is not difficult to find that it is equivalent to the CN method^10^ for these pairs of nodes with common neighbors, but distance plays an important role in predicting missing links between nodes with no common neighbors because high score is obtained by short distance. Our method is summarized in Methods section.

### Experiment

In Table 3, the predicted results of different methods under the *AUC* metric are listed in 8 real-world networks. Our method is compared with prediction methods based on common neighbors^10^: CN method, Sal (Salton Index), Jaccard Index, Sen (S*ϕ*rensen Index), HPI (Hub Promoted Index), HDI (Hub Depressed Index), LHN (Leicht-Holme-Newman Index), AA (Adamic-Adar Index) and RA method (Resource Allocation Index). For computation in these methods can be seen in Table 4. The results are the average of 20 realizations for each network under 10% and 20% probe set. Probe set will be randomly removed every time. The highest value of *AUC* for each network is labeled in boldface. The accuracy of our method outperforms other methods except Neural network, because this network possesses high *c*_*n*_, high degree heterogeneity and negative assortativity. RA index and AA index have similar form, and thus they have nearly same scores. Circuit and Power network have low *c*_*n*_, and thus for most methods assign low *AUC* scores which are approximately 0.5 for this two networks. Conversely, our method shows better results due to distance.

**Figure 2:**
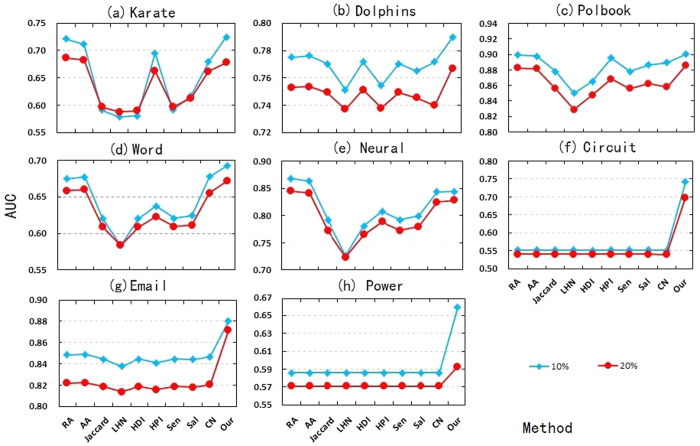
The changes of *AUC* when 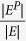 increases from 10% to 20% in 8 real-world networks (**a**–**h**).

In most algorithms, *AUC* shows slightly downward trend when the proportion of *E*^*P*^ in *E* increases from 10% to 20% (see Fig. 2). The main reason is that the decrease of training set *E*^*T*^ will result in the number of pairs of nodes with common neighbors becoming small, which increases the difficulty of link prediction.

**Figure 3:**
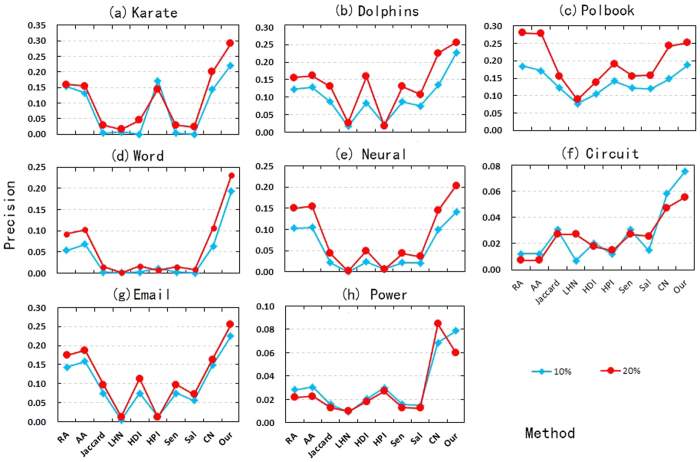
The changes of *Precision* when 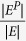 increases from 10% to 20% in 8 real-world networks (**a**–**h**).

On the other hand, the prediction results under the *Precision* metric are given in Table 5. Similarly, our method noticeably outperforms other methods except Polbook and Power network in 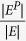 = 20%, because Power network has low 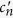 (or *c*_*n*_), high *m* and large average distance 〈*d*〉 = 18.989. According to Eq. (12) in Power network our method need predict too much missing links with large distance (i.e. the presence of few short loops with length more than 3), which result in low prediction accuracy than CN method. It should be mentioned that *Precision* indicates the opposite changing trend compared with *AUC* except Circuit and Power network (They are low 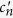) with the increase of *E*^*P*^ (see Fig. 3).

The results of most algorithms are better for high ratio of *E*^*P*^ than low one for *Precision* metric. The main reason is that the decrease of training set *E*^*T*^ will result in weak *n*′ and strong *n*′′ according to the definition of *AUC* (see Methods section), which make negative contributions to *AUC*. But the increase of probe set *E*^*P*^, the probability to obtain relevant items will increase, and it is easier to find the missing links, which is a good explanation for this phenomenon. Therefore, in practical application it is necessary to combine two metrics to evaluate accuracy of a link prediction method.

**Figure 4:**
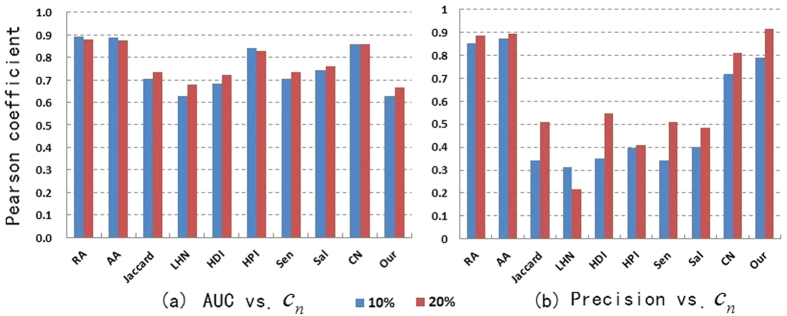
Pearson correlation coefficient of different methods for prediction accuracy metrics vs. *c*_*n*_ under 10% and 20% probe set in 8 networks. (**a**) The correlation coefficient of different methods for *AUC* metric vs. *c*_*n*_. (**b**) The correlation coefficient of different methods for *Precision* metric vs. *c*_*n*_.

Moreover, CN coefficient *c*_*n*_ of original network may also affect prediction accuracy. In Fig. 4 *AUC* metric and *c*_*n*_ have high positive correlation for almost all methods, but for *Precision* metric there are only RA, AA, CN and our method keeping high positive correlation. The change in probe set has little effect on all methods according to *AUC* metric, but makes great effect on Jaccard, HDI and Sen method according to *Precision* metric.

Next, we compared our method with other two classic methods PA (Preferential Attachment Index)^31^ and LP (Local Path Index)^11^, which do not directly relate to common neighbors, but based on local information. Table 6 indicates the prediction accuracy of LP, PA and our method under the *Precision* metric with 10% and 20% probe set, where our method has the best performance.

The most striking feature of our method is to make remarkable effect to predict missing links between nodes with no common neighbors (i.e. the accuracy to find 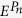), compared with LP and PA method according to *Precision* metric (see Table 7). The above mentioned methods based on common neighbors cannot find any missing links between nodes with no common neighbors, and thus we do not list them here. The results indicate that LP cannot find any missing links with respect to 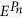, and PA method could find a small amount of 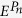 for certain networks because the proportion of 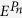 in *E*^*P*^ is low except Circuit network and Power network. The proportion can be calculated by Eq. (12).

The time-consuming of computing distance between all pairs of nodes is at most *O*(*mN*) by using BFS (Breadth First Search). For a sparse network (*m* = *O*(*N*)) the complexity of our method is equivalent to the complexity of CN method.

## Discussion and Conclusion

In the past few years, many link prediction algorithms have been proposed, but most of the algorithms do not account for missing links between two nodes with no common neighbors. Generally, to predict these missing links between nodes with no common neighbors are very difficult because they account for low proportion in missing links, but they have significance in determining network structure and network properties. We proposed in this paper a new algorithm based on common neighbors and distance to improve prediction accuracy, which separates link prediction into two parts: predicted links that generate loops of length 3 (missing links between nodes with common neighbors) and predicted links that generate short loops of length more than 3 (missing links between nodes with no common neighbors). By estimating the proportion of missing links between nodes with no common neighbors in total missing links, our algorithm makes remarkable effect to predict missing links between nodes with no common neighbors. For other methods based on common neighbors cannot find any missing links between nodes with no common neighbors. A series of experimental results indicate that prediction accuracy of our proposed method is better than most existing currently used methods for a variety of real-world networks. The complexity of this method is almost same as that of CN method. Moreover, there are two rules: (i) With the increase of probe set, experimental results indicate that the changing trend of scores according to *AUC* metric is not consistent with *Precision* metric for most algorithms and networks. Thus, it is necessary to combine two metrics to evaluate the accuracy of a link prediction method. (ii) The between *AUC* metric and *c*_*n*_ has high positive correlation.

On the other hand, the questions of link prediction have not been solved completely. For example, how to evaluate superiority of a link prediction method except current metrics. Because a good prediction method should not only take into account prediction accuracy, but also pay attention to network properties. Furthermore, it is important how to choose a suitable link prediction method according to the feature of network as there is no absolutely good method for all networks. Link prediction has been extended to weighted and directed version^32^,^33^,^34^,^35^. It can also predict signed links with positive and negative relationships in social networks, and predict spurious interactions^15^,^36^,^37^. Whether it is possible to modify our method to deal with them. All are a long-standing challenge work.

## Methods

### Metrics

*AUC* is defined as:


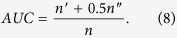


A link prediction method provides an ordered list of scores of all links in *U* − *E*^*T*^ (scores represent the likelihood of missing links), where *U* is a universal set for 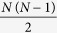 links. At each time, we will randomly select a link in *U* − *E* and a link in probe set *E*^*P*^ to compare their scores. After comparison of *n* times, there are *n*′ times the links in *E*^*P*^ having higher scores and *n*′′ times they have same scores. The degree to which the score exceeds 0.5 represents how better the method performs than pure chance.

Another metric to measure accuracy is *Precision*, which is computed as follows:


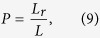


where *L*_*r*_ is relevant links (i.e. generally, we take the top-*L* links as the predicted links according to scores, and there are *L*_*r*_ links in the probe set *E*^*P*^ (*L* = |*E*^*P*^|)). Thus, the higher *Precision* value means the higher accuracy.

### Our link prediction method

Given an undirected and unweighted network *G* = (*V*, *E*) with vertex set *V* = {*v*_1_, *v*_2_, …, *v*_*N*_} and the observed link set *E*, where the size of *E* is *m*. The self-loops and multiple links are not allowed. In order to evaluate the performance of an algorithm, a certain proportion links in *G* will be randomly selected to constitute probe set *E*^*P*^, and the rest links constitute training set *E*^*T*^ (*E*^*T*^ ∪ *E*^*P*^ = *E*, *E*^*T*^ ∩ *E*^*P*^ = ∅). We separate *E*^*P*^ into two subsets: 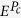 and 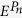 denote respectively link set between nodes with common neighbors and link set between nodes with no common neighbors 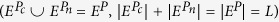. 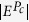 and 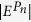 are calculated using Eqs (11 and 12) because *E*^*P*^ is used for testing and is viewed as unknown information. According to computation of *s*_*ij*_ (Eq. (13)) to obtain the scores for all non-exist links *U* − *E*^*T*^, sort the list of scores in non-increasing order. Then select top-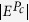 links between nodes with common neighbors and top-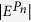 links between nodes with no common neighbors from *U* − *E*^*T*^ to constitute predicted links.


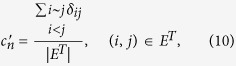



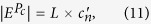



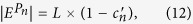


where Γ(*i*) denotes the set of neighbors of node *i*. *δ*_*ij*_ = 1, if Γ(*i*) ∩ Γ(*j*) ≠ ∅, 0 otherwise, and *i* ~ *j* denotes node *i* and *j* to be adjacent.


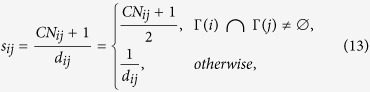


where *CN*_*ij*_ = |Γ(*i*) ∩ Γ(*j*)| is the number of common neighbors of node *i* and *j*. *d*_*ij*_ is the distance between *i* and *j*. The *Precision* metric in Eq. (9) can be written as follows:


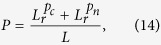


where 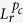, 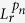 denote relevant links between nodes with common neighbors and relevant links between nodes with no common neighbors, respectively. Similarly, to predict future missing links for a current observed network *G*, 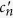 is replaced by *c*_*n*_ as follows:


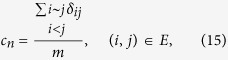


which is the proportion of links between nodes with common neighbors in link set *E*.

### Parameters

The local clustering coefficient *c*(*i*) of a node *i* is defined as the probability that two distinct neighbors of *i* are connected^38^.


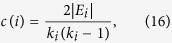


where |*E*_*i*_| denotes the number of links that actually exist between *k*_*i*_ nodes, and *c*(*i*) = 0 if *k*_*i*_ = 0, 1. The clustering coefficient *c* of a network is the average of all nodes:


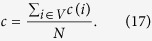


Assortativity of network is called as assortative mixing, which refers to the tendency of network nodes to joint other nodes preferentially with similar or opposite properties^39^:


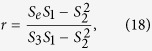


where 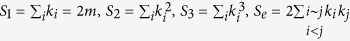.

## Additional Information

**How to cite this article**: Yang, J. and Zhang, X.-D. Predicting missing links in complex networks based on common neighbors and distance. *Sci. Rep.* **6**, 38208; doi: 10.1038/srep38208 (2016).

**Publisher's note:** Springer Nature remains neutral with regard to jurisdictional claims in published maps and institutional affiliations.

## Figures and Tables

**Table 1:** Illustration of properties of networks.

Networks	*N*	*m*	*c*	*c*_*n*_	*c*_*r*_	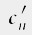	〈*d*〉	〈*k*〉	*r*	*H*
Karate	34	78	0.571	0.859	0.793	0.771	2.408	4.588	−0.476	1.693
Dolphins	62	159	0.259	0.761	0.710	0.715	3.357	5.129	−0.044	1.327
Polbook	105	441	0.488	0.959	0.937	0.927	3.079	8.400	−0.128	1.421
Word	112	425	0.173	0.725	0.694	0.672	2.536	7.589	−0.129	1.815
Neural	297	2148	0.292	0.945	0.913	0.916	2.455	14.465	−0.163	1.801
Circuit	512	819	0.055	0.137	0.118	0.115	6.858	3.199	−0.030	1.259
Email	1133	5451	0.220	0.776	0.734	0.733	3.606	9.622	0.078	1.942
Power	4941	6594	0.080	0.208	0.179	0.176	18.989	2.669	0.003	1.450

Parameters are measured in original networks *G* except 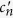 and *c*_*r*_ in *G*′ = *G* − *E*^*P*^, where 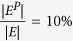.

*c*_*n*_: CN coefficient; 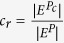; 〈*d*〉: average distance; 〈*k*〉: average degree; *c*: clustering coefficient; *r*: assortativity coefficient (see Methods section); 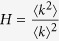: degree heterogeneity. The values of *c*_*r*_ and 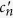 are the average of 20 realizations to randomly remove *E*^*P*^ for each network every time.

**Table 2:** Root mean square error (*EMSE*) and Pearson correlation coefficient (*CC*) between *c*_*n*_, 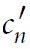 , *c*_*r*_ and clustering coefficient *c* in the 8 networks.

	*c*_*r*_	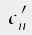		*c*_*n*_	*c*_*r*_	*c*	*c*_*n*_	*c*_*r*_	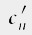
*EMSE*	0.012		*CC*	0.999		0.777		0.999	

**Table 3:** The *AUC* of different methods under 10% and 20% probe set in 8 networks.

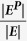	Methods	Karate	Dolphins	Polbook	Word	Neural	Circuit	Email	Power
10%	RA	0.721(78)	0.775(71)	0.899(25)	0.675(38)	**0.868(12)**	0.552(13)	0.848(11)	0.586(5)
	AA	0.711(75)	0.776(71)	0.898(25)	0.677(41)	0.863(12)	0.552(13)	0.849(11)	0.586(5)
	Jaccard	0.591(63)	0.770(67)	0.878(25)	0.621(36)	0.792(11)	0.552(13)	0.845(11)	0.586(5)
	LHN	0.578(74)	0.751(62)	0.850(26)	0.584(31)	0.727(10)	0.552(13)	0.838(10)	0.586(5)
	HDI	0.581(64)	0.772(69)	0.865(23)	0.620(35)	0.781(12)	0.552(13)	0.844(11)	0.586(5)
	HPI	0.696(79)	0.754(63)	0.895(26)	0.637(37)	0.808(12)	0.552(13)	0.841(10)	0.586(5)
	Sen	0.591(63)	0.770(67)	0.878(25)	0.621(36)	0.792(11)	0.552(13)	0.845(11)	0.586(5)
	Sal	0.617(69)	0.765(66)	0.886(25)	0.624(36)	0.800(10)	0.552(13)	0.844(11)	0.586(5)
	CN	0.679(72)	0.772(70)	0.889(26)	0.678(42)	0.844(13)	0.552(13)	0.847(11)	0.586(5)
	Our	**0.725(88)**	**0.790(60)**	**0.901(14)**	**0.693(46)**	0.844(10)	**0.742(23)**	**0.880(9)**	**0.659(10)**
20%	RA	**0.686(60)**	0.753(39)	0.883(18)	0.658(22)	**0.845(10)**	0.541(10)	0.822(6)	0.571(2)
	AA	0.683(59)	0.754(39)	0.881(18)	0.660(22)	0.842(10)	0.541(10)	0.822(6)	0.571(2)
	Jaccard	0.597(41)	0.749(37)	0.856(17)	0.609(19)	0.773(9)	0.541(10)	0.819(6)	0.571(2)
	LHN	0.588(50)	0.737(36)	0.829(19)	0.583(18)	0.724(8)	0.541(10)	0.813(6)	0.571(2)
	HDI	0.590(37)	0.751(38)	0.847(16)	0.609(19)	0.765(9)	0.541(10)	0.819(6)	0.571(2)
	HPI	0.662(63)	0.738(35)	0.868(20)	0.623(21)	0.789(10)	0.541(10)	0.816(6)	0.571(2)
	Sen	0.597(41)	0.749(37)	0.856(17)	0.609(19)	0.773(9)	0.541(10)	0.819(6)	0.571(2)
	Sal	0.613(47)	0.746(36)	0.862(18)	0.611(20)	0.780(10)	0.541(10)	0.818(6)	0.571(2)
	CN	0.662(52)	0.740(37)	0.858(17)	0.655(21)	0.824(10)	0.540(10)	0.821(6)	0.571(2)
	Our	0.678(71)	**0.767(59)**	**0.885(17)**	**0.672(20)**	0.828(6)	**0.698(23)**	**0.871(6)**	**0.593(11)**

The results are the average of 20 realizations for each network, and probe set *E*^*P*^ will be randomly removed every time. The highest value for each network is labeled in boldface. The numbers in the brackets denote the standard deviations. For example, 0.721(78) denotes that the *AUC* value is 0.721 and the standard deviation is 78 × 10^−4^.

**Table 4:** The computation of link prediction methods.

CN	*s*_*ij*_ = |Γ(*i*) ∩ Γ(*j*)|	Sal (Salton)	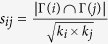
Jaccard	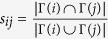	Sen (S*ϕ*rensen)	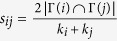
HPI (Hub Promoted)	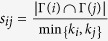	HDI (Hub Depressed)	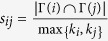
LHN (Leicht-Holme-Newman)	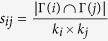	AA (Adamic-Adar)	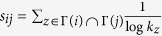
RA (Resource Allocation)	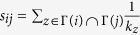		
PA (Preferential Attachment)	*s*_*ij*_ = *k*_*i*_ × *k*_*j*_	LP (Local Path)	*s*^*LP*^ = *A*^2^ + *βA*^3^

*k*_*i*_ is the degree of node *i*. PA method and LP method do not directly relate to common neighbors, but based on local information, where *A* is adjacency matrix and *β* = 0.01.

**Table 5:** The *Precision* of different methods under 10% and 20% probe set in 8 networks.

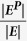	Methods	Karate	Dolphins	Polbook	Word	Neural	Circuit	Email	Power
10%	RA	0.154(109)	0.123(73)	0.185(55)	0.054(27)	0.103(17)	0.012(9)	0.143(12)	0.028(9)
	AA	0.132(125)	0.128(81)	0.172(53)	0.068(39)	0.105(20)	0.012(9)	0.158(13)	0.031(8)
	Jaccard	0.004(16)	0.087(58)	0.122(46)	0.002(4)	0.021(10)	0.031(18)	0.074(9)	0.016(2)
	LHN	0.007(22)	0.017(30)	0.077(48)	0.001(5)	0.000(1)	0.007(9)	0.004(3)	0.009(2)
	HDI	0.000(0)	0.083(64)	0.105(45)	0.002(7)	0.023(8)	0.020(12)	0.075(9)	0.020(1)
	HPI	0.171(91)	0.022(25)	0.142(52)	0.011(9)	0.007(4)	0.012(9)	0.013(4)	0.030(3)
	Sen	0.004(16)	0.087(58)	0.122(46)	0.002(4)	0.021(10)	0.031(18)	0.074(9)	0.016(2)
	Sal	0.000(0)	0.075(57)	0.120(39)	0.000(0)	0.021(10)	0.015(15)	0.056(8)	0.015(2)
	CN	0.143(73)	0.135(57)	0.148(46)	0.063(32)	0.099(20)	0.058(21)	0.149(15)	0.069(18)
	Our	**0.221(95)**	**0.227(55)**	**0.188(46)**	**0.193(33)**	**0.141(19)**	**0.075(17)**	**0.225(17)**	**0.079(4)**
20%	RA	0.160(76)	0.156(38)	**0.280(22)**	0.092(33)	0.150(12)	0.007(4)	0.174(9)	0.022(3)
	AA	0.155(78)	0.161(43)	0.278(32)	0.102(31)	0.155(14)	0.007(4)	0.188(9)	0.023(2)
	Jaccard	0.028(50)	0.131(53)	0.157(34)	0.014(17)	0.043(10)	0.027(13)	0.097(5)	0.013(4)
	LHN	0.015(23)	0.027(25)	0.089(27)	0.001(3)	0.002(2)	0.027(12)	0.012(3)	0.010(3)
	HDI	0.045(47)	0.159(47)	0.138(35)	0.017(18)	0.050(10)	0.018(7)	0.113(7)	0.018(4)
	HPI	0.145(70)	0.018(14)	0.192(36)	0.006(6)	0.006(2)	0.015(7)	0.012(2)	0.027(3)
	Sen	0.028(50)	0.131(53)	0.157(34)	0.014(17)	0.043(10)	0.027(13)	0.097(5)	0.013(4)
	Sal	0.023(42)	0.108(47)	0.158(33)	0.009(12)	0.036(9)	0.025(11)	0.071(8)	0.013(4)
	CN	0.200(59)	0.226(44)	0.243(59)	0.107(30)	0.146(13)	0.047(9)	0.163(16)	**0.085(5)**
	Our	**0.292(101)**	**0.256(64)**	0.252(44)	**0.231(32)**	**0.203(15)**	**0.056(11)**	**0.255(5)**	0.060(4)

The results are the average of 20 realizations for each network, and probe set *E*^*P*^ will be randomly removed every time. The highest value for each network is labeled in boldface. The numbers in the brackets denote the standard deviations. For example, 0.154(109) denotes that the *Precision* value is 0.154 and the standard deviation is 109 × 10^−4^.

**Table 6:** The *Precision* of LP, PA and our method under 10% and 20% probe set in 8 networks.

Networks	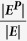	PA	LP	Our	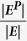	PA	LP	Our
Karate	10%	0.068(82)	0.175(140)	**0.221(95)**	20%	0.118(70)	0.177(88)	**0.292(101)**
Dolphins		0.020(29)	0.133(70)	**0.227(55)**		0.025(31)	0.199(47)	**0.256(64)**
Polbook		0.044(32)	0.172(44)	**0.188(46)**		0.088(25)	0.221(39)	**0.252(44)**
Word		0.082(43)	0.083(31)	**0.193(33)**		0.150(32)	0.102(30)	**0.231(32)**
Neural		0.054(18)	0.099(18)	**0.141(19)**		0.098(9)	0.145(14)	**0.203(15)**
Circuit		0.002(4)	0.005(7)	**0.075(17)**		0.003(3)	0.020(8)	**0.056(11)**
Email		0.018(7)	0.136(11)	**0.225(17)**		0.029(5)	0.175(10)	**0.255(5)**
Power		0.001(1)	0.042(2)	**0.079(4)**		0.002(1)	0.045(6)	**0.060(4)**

The results are the average of 20 realizations for each network, and probe set will be randomly removed every time. The highest value is labeled in boldface. The numbers in the brackets denote the standard deviations. For example, 0.068(82) denotes that the *Precision* value is 0.068 and the standard deviation is 82 × 10^−4^.

**Table 7:** The *Precision* of different methods to predict missing links between nodes with no common neighbors under 10% and 20% probe set in 8 networks.

Networks	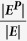	PA	LP	Our	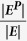	PA	LP	Our
Karate	10%	0.05(224)	0(0)	**0.353(207)**	20%	0.064(116)	0(0)	**0.351(169)**
Dolphins		0(0)	0(0)	**0.267(129)**		0.004(19)	0(0)	**0.265(70)**
Polbook		0(0)	0(0)	**0.432(104)**		0(0)	0(0)	**0.427(47)**
Word		0(0)	0(0)	**0.430(54)**		0.003(9)	0(0)	**0.410(36)**
Neural		0(0)	0(0)	**0.441(25)**		0.005(8)	0(0)	**0.457(20)**
Circuit		0.002(5)	0(0)	**0.081(17)**		0.004(3)	0(0)	**0.059(12)**
Email		0.001(2)	0(0)	**0.340(21)**		0.004(4)	0(0)	**0.335(9)**
Power		0(1)	0(0)	**0.059(5)**		0(0)	0(0)	**0.047(3)**

*Precision* = 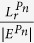, which denotes the proportion of relevant links in the probe set 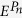. The results are the average of 20 realizations for each network, and probe set *E*^*P*^ will be randomly removed every time. The highest value for each network is labeled in boldface. The numbers in the brackets denote the standard deviations. For example, 0.064 (116) denotes that the *Precision* value is 0.064 and the standard deviation is 116 × 10^−4^. The previous mentioned methods based on common neighbors cannot find any missing links between nodes with no common neighbors, and thus we do not list them here.
